# PHLPP1 deficiency alleviates dopaminergic neurodegeneration and represses neuroinflammation in Parkinson’s disease

**DOI:** 10.1186/s12993-025-00293-y

**Published:** 2025-09-29

**Authors:** Zhilin Chen, Yuan Liu, Jinyue Zhao, Xin Zhou, Yudi Han, Zikai Zhou, Huazheng Liang, Yong Bi

**Affiliations:** 1 Department of Neurology, Zhoupu Hospital affiliated to Shanghai University of Medicine & Health Sciences, 1500 Zhouyuan Road, Pudong New Area, Shanghai, 201318 China; 2 Department of Neurology, Shanghai Tongji Hospital, School of Medicine, Tongji University, Shanghai, China; 3 Translational Research Institute of Brain and Brain-Like Intelligence, Shanghai Fourth People’s Hospital, School of Medicine, Tongji University, Shanghai, China; 4 Zhongshan Institute for Drug Discovery, Shanghai Institute of Materia Medica, Chinese Academy of Sciences, Zhongshan, China; 5 Monash Suzhou Research Institute, Suzhou, Jiangsu Province China

**Keywords:** Pleckstrin homology domain leucine-rich repeat protein phosphatases (PHLPP), Parkinson’s disease, Neuroinflammation, NLRP3, Caspase1, IL-1β, Proinflammatory cytokines, Anti-inflammatory cytokines

## Abstract

**Background:**

Pleckstrin homology (PH) domain leucine-rich repeat protein phosphatases (PHLPP) has been associated with several neurodegenerative diseases, however, few studies have investigated the role of PHLPP in Parkinson’s disease (PD). The present study aimed to answer this question through establishing a Parkinson’s disease (PD) model using the Phlpp1-/- and wild-type (WT) mice and testing their behavioral as well as molecular changes. Methods: MPTP was intraperitoneal injected into mice to generate a PD model. Neurobehavioral parameters, protein expression and inflammatory cytokines release were measured by the open filed test, the pole test, immunohistochemistry, immunoblotting, immunoprecipitation, and quantitative reverse transcription PCR.

**Results:**

MPTP-induced neurobehavioral deficits were more significantly ameliorated in PHLPP-KO-MPTP mice compared to WT-MPTP mice. The survival rate of TH^+^ neurons in the PHLPP-KO-MPTP group was higher than that in the WT-MPTP group (66% vs. 38%). Additionally, PHLPP1 knockout in KO-MPTP mice markedly reduced levels of IL-1β, IL-6, TNF-α, and iNOS, and increased levels of TGF-β compared to those of WT-MPTP mice. Furthermore, PHLPP1 was found to bind to NLRP3 and that PHLPP1 knockout inhibited MPTP-induced expression of IL-1β and caspase-1 in substantia nigra of PD model mice.

**Conclusion:**

Our results demonstrates that PHLPP1 knockout in PD model is positively associated with the survival of TH + neurons by suppressing inflammatory response in substantia nigra, suggesting that PHLPP1 plays a critical role in the development of PD.

## Background

Parkinson’s Disease (PD), the second most common neurodegenerative condition after Alzheimer’s disease, particularly among the elderly. The hallmark symptoms of PD include tremors, movement difficulties, and problems with balance and coordination [1]. Currently, PD diagnosis is primarily relying on clinical symptoms; however, the gold standard for diagnosis is based on histopathological features, which reveal dopaminergic neuronal loss and the aggregation of Lewy bodies in the cytoplasm of neurons of substantia nigra (SN), the dorsal nucleus of the vagus nerve, the cerebral cortex, the intestinal myenteric plexus or sympathetic ganglia [2, 3]. Aggregated α-synuclein is the key component of Lewy bodies, which are neuronal inclusions present in SN of PD patients [4–6]. Dysfunction of dopaminergic neurons in SN can trigger neuroinflammation in PD. Similar to other neurodegenerative diseases, neuroinflammation plays an important role in PD and is predominantly mediated by microglia and their secreted inflammatory cytokines. Various forms of defective α-synuclein (monomeric, oligomeric, or fibrillar) can interact with membrane receptors on microglia, leading to their activation and the release of pro-inflammatory cytokines, such as IL-1β, IL-6, IL-18, IL-23,TNF-α, IFN-γ, nitric oxide (NO), and reactive oxygen species (ROS) [7–11].

Pleckstrin homology (PH) domain leucine-rich repeat protein phosphatases (PHLPP) are newly identified members of the type 2 C phosphatase (PP2C) family of serine/threonine phosphatases, which belong to the metal-dependent (PPM) phosphatase family. PHLPP comprises two isoforms: PHLPP1 (which includes PHLPP1α and PHLPP1β/SCOP) and PHLPP2. Both isoforms have a C-terminal PDZ binding motif, an N-terminal pleckstrin homology (PH) domain, a protein phosphatase (PP2C) domain, and a series of leucine-rich repeats (LRR). PHLPP are expressed in various compartments of cells, including the plasma membrane, cytoplasm, nucleus, and mitochondria. Its regulatory domains enhance substrate specificity through interactions with scaffolding proteins, contributing to its versatility [12]. PHLPP are present in all body tissues and particularly abundant in the brain. Recent studies have provided evidence for the expression of both PHLPP isoforms in neurons and astrocytes [13]. PHLPP are implicated in neuronal survival, apoptosis, memory formation, and memory consolidation [14]. Their effects are primarily mediated through substrates, such as AKT, PKC, and ERK within the brain tissue.

A number of studies have indicated that inhibiting PHLPP1 leads to the phosphorylation of AKT, which plays a neuroprotective role. For instance, Chen et al. found that PHLPP1 knockout (KO) mice exhibited significantly increased Akt activity following middle cerebral artery occlusion (MCAO) for 2 h, along with reduced neurovascular damage after reperfusion. The infarct size of PHLPP1-KO mice was significantly smaller, and astrocytes as well as neurons in PHLPP1-KO mice showed increased Akt activation and reduced cell death under hypoxia-glucose deprivation when PHLPP1 was down-regulated [14]. These findings align with those of Jackson et al. who reported that PHLPP1 knockdown in primary cortical neurons of rats increased basal levels of Akt and phosphorylation levels of Extracellular signal-regulated kinase (ERK) as well as neuronal viability during staurosporine (STS)-induced cell death [15].

ERK1/2 plays a key role in various forms of neuroplasticity, including memory formation and long-term memory consolidation. Activation of ERK1/2 regulates the expression of the cAMP response element (CRE), and it is known that PHLPP1β/SCOP inhibits transcription of CRE downstream genes in cultured primary hippocampal neurons. Shimizu et al. observed that overexpression of PHLPP1β/SCOP exerted minimal impact on short-term memory (assessed 8 min post-training) but significantly impaired long-term memory (assessed 24 h post-training) [16].

Overall, PHLPP1 plays a key regulatory role in neurons through its interactions with various substrates, and it is associated with diverse neurodegenerative diseases, including Alzheimer’s disease [17] and Huntington’s disease [18]. However, the role of PHLPP1 in PD remains unclear.

To date, there are few studies investigating the mechanistic role of PHLPP1 in PD. While inhibition of PHLPP1 protects neurons via the AKT pathway, its effects on dopaminergic neurons in the SN of Parkinson’s disease patients remain unexplored. Moreover, although PHLPP1 is involved in the AKT regulatory pathway in astrocytes, which is critical in the context of neuroinflammation in PD [7, 15], its specific effects on neuroinflammation in PD have yet to be clarified. Results of this study suggest that PHLPP1 may influence the survival of dopaminegic neurons by modulating the inflammatory immune environment within the SN. The present study aimed to demonstrate whether PHLPP1 deficiency in a PD model can significantly protect against neurodegeneration and its potential mechanisms. PD model was made by injecting MPTP into the peritoneum and behavioural as well as molecular biology techniques were employed to answer the questions.

## Methods

### Animals

Phlpp1 knockout (Phlpp1-/-) mice were generated using a geneome editing technique CRISPR-Cas9 (Cyagen Biosciences, Suzhou, China). Exon 2 of the Phlpp1 gene (NCBI Reference Sequence: NM_133821) was identified as the target region for conditional knockout. The disruption of exon 2 results in a frameshift mutation in the Phlpp1 gene, leading to the loss of PHLPP1 function. Wild-type (WT) mice (C57BL/6 strain) were obtained from the Shanghai SLAG Laboratory Animal Corporation (Shanghai, China). All mice used in the study were 10–12 weeks old and weighed between 25 and 30 g. The animals were housed in a well maintained environment by setting the temperature to 22 ± 1 °C with the humidity setting of 50–60%, and the lighting was set on a 12-hour light/dark cycle. Mice can access food and water ad libitum. After they arrive at the animal facililty, they will have 7 days to acclimate to the new environment.

The present study was conducted by complying with the guideline established by the National Institutes of Health (publication No. 80 − 23). Animal ethics approval was granted by the Animal Care and Ethics Committee of Tongji University.

### Parkinson’s disease modeling

**Fig. 1:**
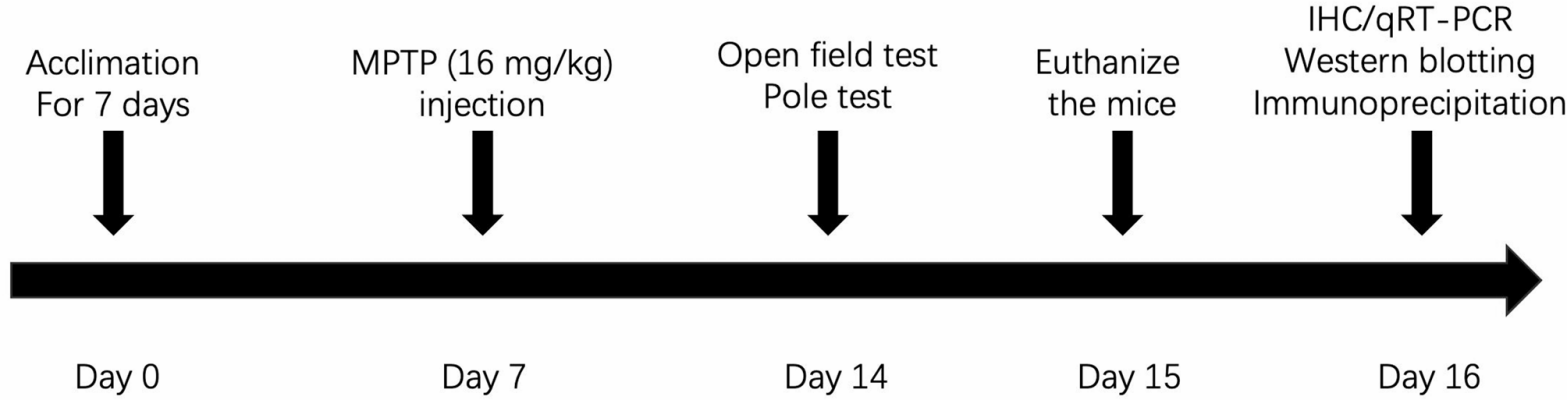
Experimental design. Mice were administered four consecutive intraperitoneal (i.p.) injections of MPTP (16 mg/kg) or 0.9% saline with 2-hour intervals after 7-day acclimation in the animal house. Seven days after the final MPTP injection, behavioral testing was performed and the next day mice were euthanized for tissue collection. Subsequently immunohistochemistry (IHC), Western blotting, immunoprecipitation, and quantitative reverse transcription PCR (qRT-PCR) were conducted

To establish a PD model, male mice received four consecutive intraperitoneal (i.p.) injections of MPTP (16 mg/kg; Sigma-Aldrich Chemical, MO, USA) with 2-hour intervals [19–21]. Mice in the control group received the same volume of saline as the model mice. Mice underwent behavioral tests 7 days after the final MPTP injection (day 14)(Fig. 1).

### The open field test

Motor behavior was assessed using the open field test 7 days post-MPTP administration. The open field test is a widely utilized method for evaluating spontaneous locomotor activity in PD model [22]. It consists of a square arena surrounded by walls (40 × 40 × 30 cm), and an infrared beam-break sensors was set above the center of the arena to monitor animal movement. The arena was divided into 16 squares by gridlines. Mice were placed in the center before the experiment started, and their behavior was recorded. The experimenter who sat 1 m away recorded the following parameters using the SuperMaze V2.0 software (Shanghai Xin Ruan, China) over a period of 5 min: the total distance traveled, the mean speed, the number of rearing events, and gridline crossings.

### The pole test

The pole test is an established method for evaluating bradykinesia in PD mouse models. This test was performed 7 days after the final MPTP injection, following the protocol by Wu et al. [23]. A pole of 55 cm long and 10 mm wide was used. Mice were placed on the top of the pole with their heads facing upwards. The time taken by each mouse to descend to the bottom end of the pole was recorded. Motor performance data were presented using the mean time spent on the pole (across three trials).

### Immunohistochemistry

Mice were anesthetized with a lethal dose of anesthetic on day 15. Immunohistochemical staining was performed according to a previous method [24]. Mice were first transcardially perfused with pre-cooled phosphate-buffered saline (PBS), and then by 4% paraformaldehyde (PFA) in the phosphate buffer (0.1 M). After removal, mouse brains were fixed in 4% PFA solution for overnight and then dehydrted in 30% sucrose solution in PBS (pH 7.4). The brains were then frozen in the cryostat and cut into serial coronal Sect. (20 μm thickness).

Brain sections were first incubated in 0.3% H_2_O_2_ solution for 30 min at room temperature. Following rinsing with PBS, these sections were permeabilized with 0.5% Triton X-100 for 10 min. Afterward, sections were incubated in 5% serum from the same species as the secondary antibody for 1 h at room temperature to block non-specific binding sites, and subsequently in the primry antibody solution (rabbit anti-tyrosine hydroxylase (TH) antibody, 1:1000, Abcam, ab112) at 4 °C for overnight and secondary antibody solution (goat anti rabbit-HRP, 1:2000, Abcam, ab288151). Staining was completed using the ABC method with 3,3′-diaminobenzidine (DAB) as the peroxidase substrate. TH-positive dopaminergic neurons in SN were captured using a Leica microscope system (Wetzlar, Germany). Stereological counting of TH-positive neurons was performed using established methods from a previous study [25].

### Immunoblotting and immunoprecipitation

Mice were euthanized with overdose anesthetics 8 days after MPTP injection (day 15). Brains were immediately harvested, and SN was dissected for subsequent Western blot analyses [21]. SN samples were extracted using a tissue protein extraction kit (Thermo Fisher Scientific). Protein concentrations were tested using the BCA protein assay (a kit from Thermo Fisher Scientific). Equal amounts of protein extracts were separated on SDS-PAGE gels, and transferred onto PVDF membranes. The latter were then incubated in 5% skim milk for 1 h to block non-specific binding sites and subsequently incubated in the primary antibody(TH, 1:1000, Abcam, ab112; β-actin, 1:2000, Cell Signaling Technology, 3700; IL-1β, 1:1000, Abcam, ab9722; caspase-1, 1:1000, AdipoGen Life Sciences, AG-20B-0042-C100) solution for overnight at 4 °C. Following this, the membranes were transferred to the secondary antibody solution (HRP-conjugated) for 2 h before imaging the results.

For immunoprecipitation, 400 µg of protein extract from each group was incubated in the corresponding primary antibody (PHLPP1, 1:2000, Bethy Laboratories, A300-660 A; NLRP3, 1:1000, AdipoGen Life Sciences, AG-20B-0014-C100) solution or an IgG control for overnight at 4 °C. Protein A/G agarose beads (Santa Cruz Biotechnology) were then added to the samples and incubated for 2 h. The protein-bead mixture was then centrifuged, washed, and collected for immunoblot analysis. To minimize the interaction of secondary antibodies with the heavy or light chains of the immunoprecipitating primary antibodies, anti-mouse IgG TrueBlot or anti-Rabbit IgG TrueBlot ULTRA HRP-conjugated secondary antibodies (Rockland Immunochemicals, 18-8817-30, 18-8816-31) were employed.

### Quantitative reverse transcription PCR (qPCR)

The qPCR was performed following the previous methods [21, 26]. Total RNA was isolated from substantia nigra using TRIzol (Thermo Fisher Scientific) and reverse-transcribed to cDNA from 1 µg of total RNA using the PrimeScript RT reagent kit with gDNA Eraser (TaKaRa). qPCR was conducted on a QuantStudio 6 Flex System (Thermo Fisher Scientific) using a SYBR master mix (Toyobo, QPK-201). Data were normalized to the glyceraldehyde-3-phosphate dehydrogenase (GAPDH) expression level. Primers for the target genes were purchased from JIELI Biology (Shanghai, China), and their sequences were:

**Table:** 

IL-1β	F:5’-ATGATGGCTTATTACAGTGGCAA-3’
	R: 5’-GTCGGAGATTCGTAGCTGGA-3’
IL-6	F:5’-ACTCACCTCTTCAGAACGAATTG-3’
	R:5’-CCATCTTTGGAAGGTTCAGGTTG-3’
TNF-α	F: 5’-CCTCTCTCTAATCAGCCCTCTG-3’
	R: 5’-GAGGACCTGGGAGTAGATGAG-3’
TGF-β	F: 5’-CTCCCGTGGCTTCTAGTGC-3’
	R: GCCTTAGTTTGGACAGGATCTG-3’
iNOS	F: 5’-GTGGTGACAAGCACATTTGG-3’
	R: 5’-AAGGCCAAACACAGCATACC-3’
GAPDH	F:5′ -AGGTCGGTGTGAACGGATTTG-3′
	R:5’-TGTAGACCATGTAGTTGAGGTCA − 3’

### Statistical analysis

Statistical analyses were were conducted using the GraphPad Prism 7 software. Differences between two groups were analyzed using the unpaired Student’s t-test. For multiple comparisons between three or more groups, One-way ANOVA was employed, followed by the Student-Newman-Keuls post hoc test. Results were presented as the mean ± standard deviation (SD). *p*-value < 0.05 was considered statistically significant.

## Results

### PHLPP1 deficiency protected against MPTP-induced motor dysfunction

To explore the role of PHLPP1 deficiency in Parkinson’s disease (PD) mouse models, we utilized PHLPP1 knockout (KO) mice alongside wild-type (WT) mice subjected to 1-methyl-4-phenyl-1,2,3,6-tetrahydropyridine (MPTP), a neurotoxin that induces Parkinsonian symptoms.

**Fig. 2:**
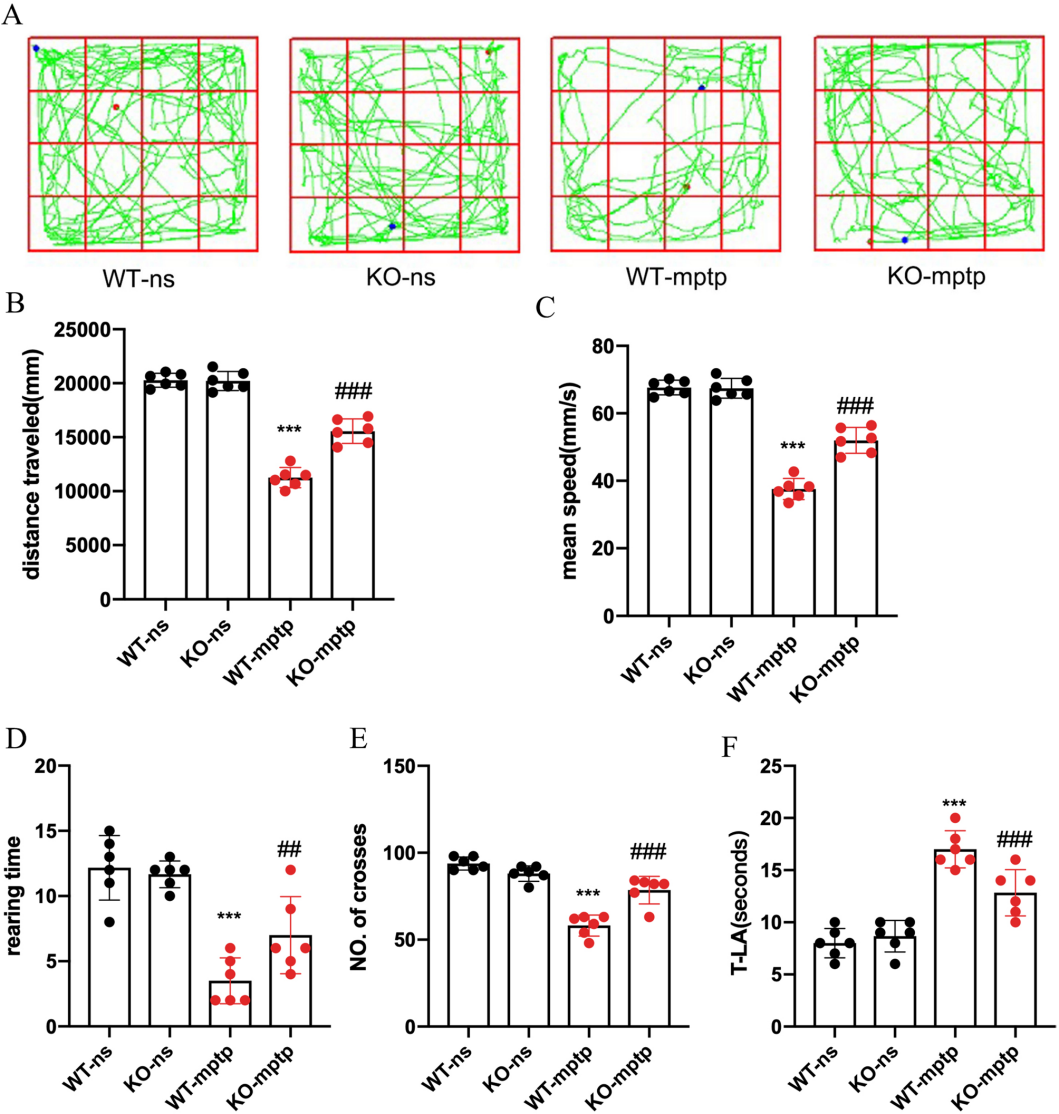
PHLPP1 deficiency ameliorates motor dysfunction in MPTP-induced mice. (**A**) Movement paths in the different experimental groups. (**B**)Total distance traveled in the different experimental groups. (**C**) Mean speed of the different experimental groups. (**D**)Rearing time in the different experimental groups. (**E**)The number of squares crossed by mice. (**F**) For the pole test, the time for every mouse to reach the bottom of the pole was recorded and analysed. Values are presented as the mean ± SD(*n* = 6 mice per group)(**B**-**G**), **p < 0.01, ****p < 0.001: WT-mptp vs. WT-ns, ^##^*p* < 0.01, ^###^*p* < 0.001: KO-mptp vs. WT-mptp

We employed two behavioral tests: the open field and the pole tests, to evaluate motor dysfunction resulting from MPTP treatment. During the open field test, we monitored movements of mice on the 7th day post-MPTP injection, and recorded metrics, such as total distance traveled over 5 min, mean speed, rearing times, and the number of crossings within the arena. In comparison to the WT-non-symptomatic (WT-ns) group, WT-MPTP mice exhibited significantly reduced activity, characterized by lower total distance traveled, decreased speed, fewer crossing events, and reduced rearing behavior (Fig. 2A-E). Conversely, KO-MPTP mice demonstrated increased activity levels relative to WT-MPTP mice, with significantly higher speed, more crossings, and greater rearing occurrences (Fig. 2A-E).

In the pole test, we evaluated the time to descend from a vertical pole. The WT-MPTP group showed a markedly prolonged descent time compared to the WT-ns group (Fig. 2F), indicating impaired motor coordination. In contrast, the KO-MPTP group significantly ran faster than the WT-MPTP group (Fig. 2F).

These findings collectively suggest that PHLPP1 deficiency appears to confer a protective effect against MPTP-induced neurobehavioral deficits, highlighting its potential role in modulating the pathophysiology of PD.

### PHLPP1 deficiency ameliorated the dopaminergic neuronal death in the SN of PD mice

**Fig. 3:**
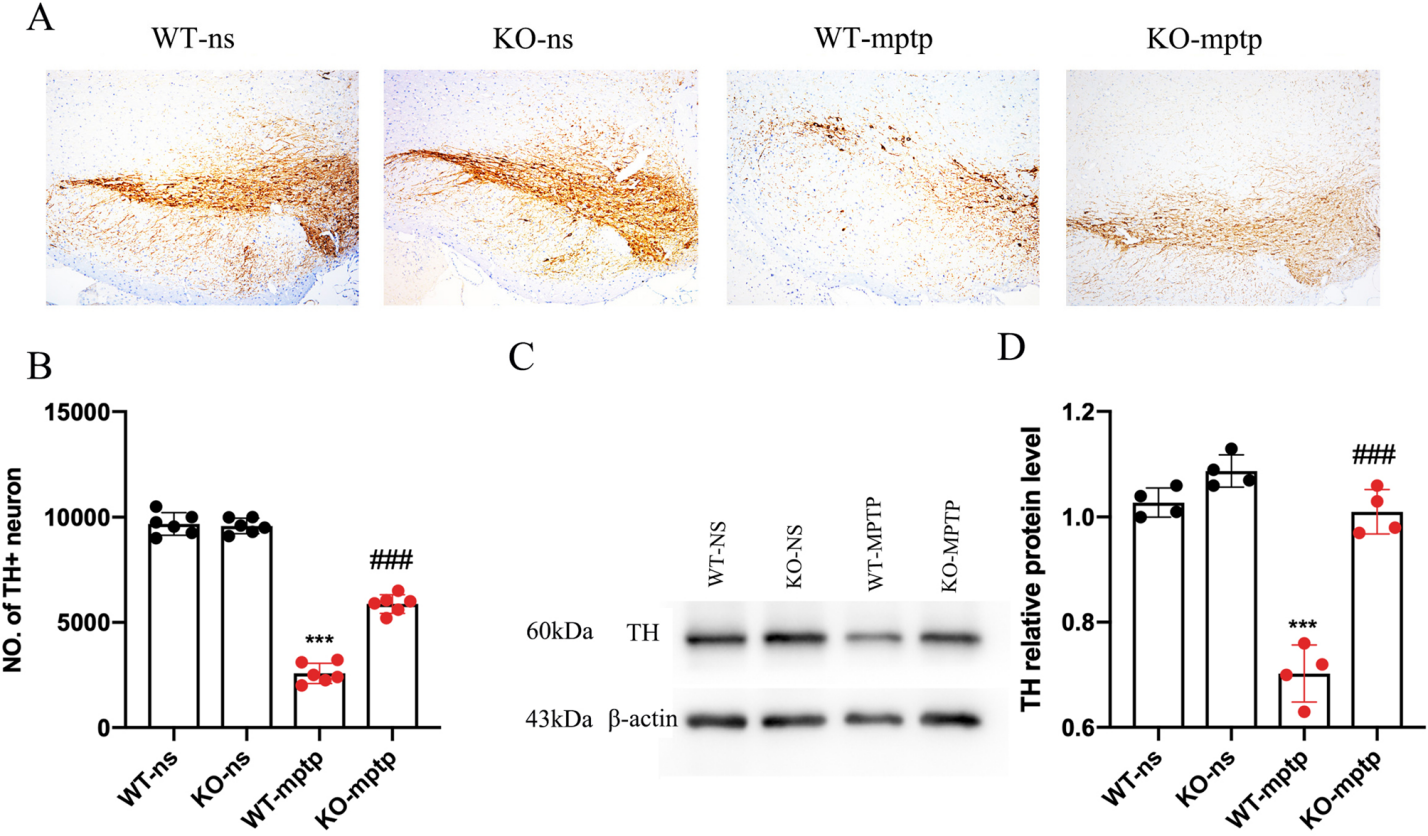
PHLPP1 deficiency reduces MPTP-induced loss of TH. (**A**) Dopaminergic neurons were evaluated by immunohistochemical of TH in the SN. (**B**) Stereology counts of the TH + neuron in the SN(*n* = 6 mice per group). (**C**, **D**) Western blotting and semiquantitative densitometry analysis of TH in SN (*n* = 4 mice per group). Values are presented as the mean ± SD, ***p < 0.001: WT-mptp vs. WT-ns; ^###^ *p* < 0.001: KO-mptp vs. WT-mptp

To assess the impact of PHLPP1 deficiency on dopaminergic neurons in the SN of PD mouse model, we compared each group using Immunohistochemistry of TH. The results showed that compared with the WT-ns group, the proportion of surviving TH + neurons in the WT-MPTP group was 38%, while in the KO-MPTP group it was 66% (*p* < 0.001; Fig. 3A, B), revealing that PHLPP1 deficiency can significantly improve the survival of TH + neurons.

Additionally, we conducted western blot analysis to evaluate the expression levels of TH in the SN. The results indicated a notable downregulation of TH expression in the SN of WT-MPTP mice vs. WT-ns mice (*p* < 0.001; Fig. 3C, D). In contrast, PHLPP1 deficiency in the KO-MPTP group mitigated the decline of TH expression compared to WT-MPTP mice (*p* < 0.001; Fig. 3C, D).

These results collectively suggest that PHLPP1 deficiency protects against MPTP-induced dopaminergic neuronal death and improves overall neurological outcomes in this PD model. This highlights the potential neuroprotective role of PHLPP1 deficiency in the context of Parkinson’s disease pathology.

### PHLPP1 deficiency alleviated inflammation in SN of PD mice

**Fig. 4:**
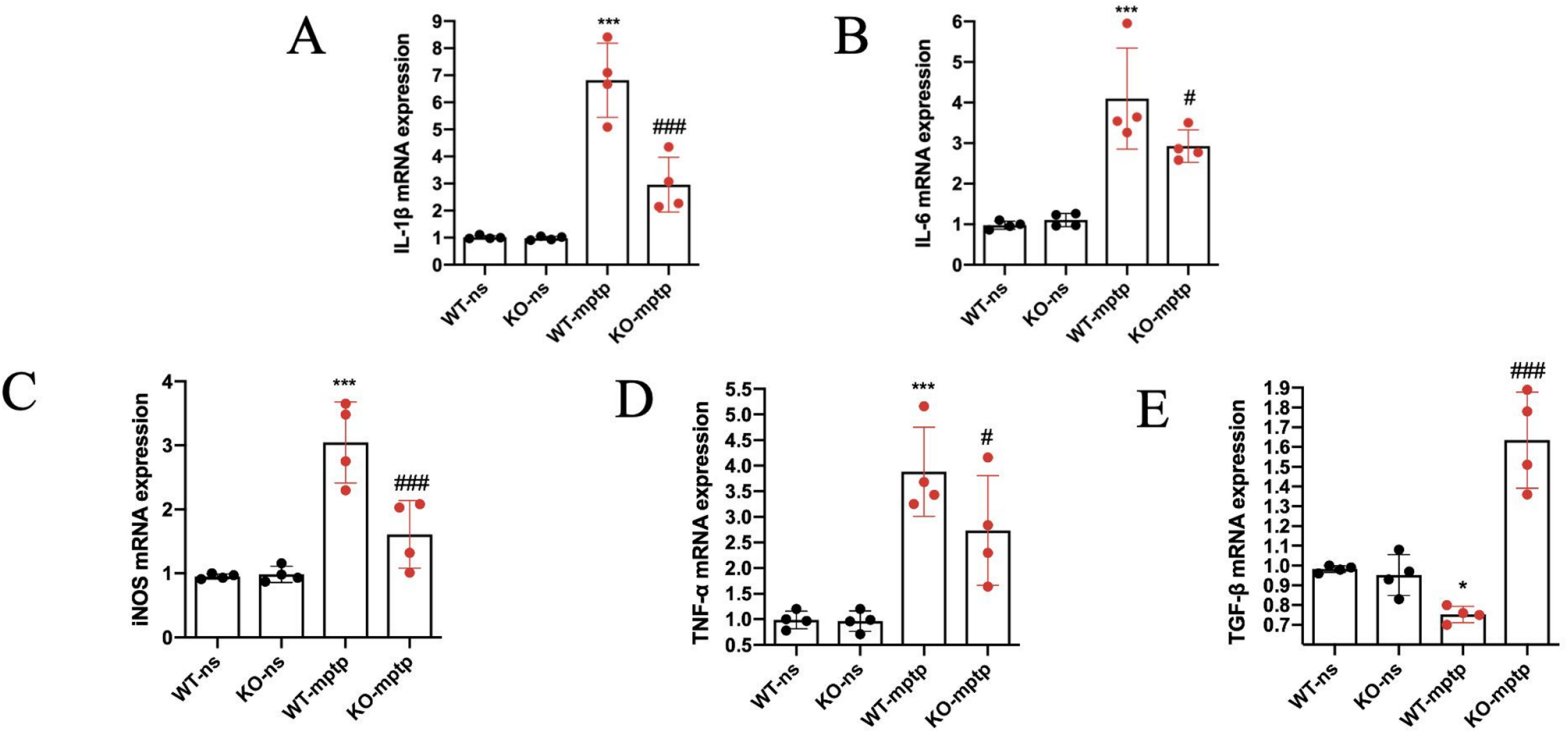
PHLPP1 deficiency decreases the expression of the inflammation- associated molecules in the SN of PD mice. (**A-E**) Q-PCR analysis of IL-1β, IL-6, TNF-α, TGF-β and iNOS mRNA expression in the SN (*n* = 4 mice per group). Values are presented as the mean ± SD, *p < 0.05, ***p < 0.001: WT-mptp vs. WT-ns; ^#^*p* < 0.05, ^###^ *p* < 0.001: KO-mptp vs. WT-mptp

We next investigated the differences in the expression of pro-inflammatory and anti-inflammatory cytokines in the SN of each group. The mRNA expression levels of several pro-inflammatory mediators, including IL-1β, IL-6, TNF-α, and iNOS, were significantly elevated in the SN of WT-MPTP mice compared to WT-ns mice (Fig. 4A-D). Conversely, the expression of the anti-inflammatory cytokine TGF-β was markedly reduced in the SN of WT-MPTP mice compared to WT-ns mice (Fig. 4E). In the KO-mptp mice, PHLPP1 deficiency markedly inhibited the expression levels of IL-1β,IL-6, TNF-α and iNOS (Fig. 4A-D), and improved the expression levels of TGF-β compared with WT-mptp mice (Fig. 4E).

Our results suggest that PHLPP1 deficiency plays a key role in modulating the inflammatory response in the SN, by reducing levels of pro-inflammatory cytokines and enhancing levels of anti-inflammatory cytokines. This modulation may contribute to the neuroprotective effect observed in KO-MPTP mice, further supporting the potential therapeutic implication of targeting PHLPP1 in Parkinson’s disease.

### PHLPP1 deficiency inhibited MPTP-Induced activation of IL-1β and caspase1 in the SN of PD mice

**Fig. 5:**
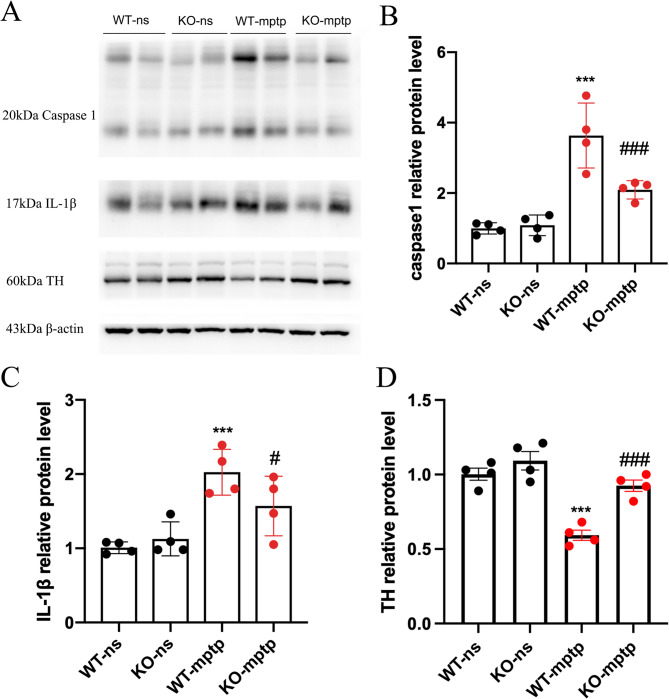
PHLPP1 deficiency inhibits MPTP-Induced activation of IL-1β and caspase1 in the SN of PD mice. (**A**) Western blotting of caspase1, IL-1β, TH and β-actin in SN. (**B-D**) Quantification analysis of caspase1 IL-1β and TH protein levels normalized to β-actin(*n* = 4 mice per group). ^***^p < 0.001: WT-mptp vs. WT-ns; ^#^*p* < 0.05, ^###^ *p* < 0.001: KO-mptp vs. WT-mptp

To confirm whether PHLPP1 deficiency suppresses the activation of IL-1β and caspase-1 in the SN of PD mice, we measured the protein levels of IL-1β and caspase-1 following MPTP treatment. Our results demonstrated that MPTP injection significantly increased the expression of both IL-1β and caspase-1 in the SN (Fig. 5A-D). Notably, PHLPP1 deficiency was found to inhibit MPTP-induced activation of IL-1β and caspase-1 in comparison to the WT-MPTP mice (Fig. 5A-D). When TH was measured at the same time, it could be seen that MPTP significantly decreased the level of TH, and PHLPP1 knockout reversed this decrease (Fig. 5A).

**Fig. 6:**
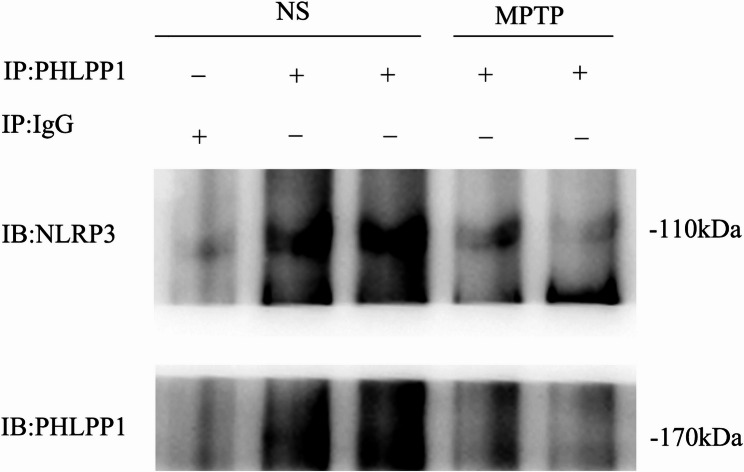
PHLPP1 interacts with NLRP3. The lysates of SN from wild-type (WT) mice subjected to saline or MPTP were immunoprecipitated with the PHLPP1 Ab and then immunoblotted with NLRP3 and PHLPP1Abs

Subsequently, we investigated the protein interactions that PHLPP1 engages to inhibit the activation of IL-1β and caspase-1. The SN lysates from WT mice that received either saline or MPTP treatment were immunoprecipitated with antibodies against PHLPP1 and then analyzed via immunoblotting for NLRP3 and PHLPP1. Our results revealed that PHLPP1 bound to NLRP3 within the SN of WT-MPTP mice (Fig. 6).

Our results suggest that PHLPP1 may play an essential role in regulating inflammatory response in PD by interacting with NLRP3, potentially inhibiting the activation pathways of IL-1β and caspase-1. This interaction may provide further insights into the mechanisms behind the neuroprotective effects observed with PHLPP1 deficiency in the context of PD.

## Discussion

Findings of this study provide compelling evidence for the role of PHLPP1 in dopaminergic neurodegeneration and neuroinflammation associated with PD. Our results demonstrated that PHLPP1 deficiency can alleviate motor dysfunction in a MPTP-induced PD mouse model by reducing the loss of TH + neurons and regulating pro-inflammatory and anti-inflammatory response in SN of PD mice.

Additionally, we uncovered a novel interaction between PHLPP1 and NLRP3. Specifically, PHLPP1 deficiency was shown to inhibit the MPTP-induced activation of IL-1β, possibly mediated through the NLRP3-PYCARD-caspase-1 signaling pathway. This inhibition suggests that PHLPP1 may play a pivotal role in regulating neuroinflammation during PD progression. Collectively, our findings highlight the potential of targeting PHLPP1 as a therapeutic node to mitigate neuroinflammation and protect dopaminergic neurons in PD, opening new avenues for future research and intervention strategies.

Boonying et al. discovered that knockdown of PHLPP could protect neurons from death due to the injection of 1-methyl-4-phenylpyridinium (MPP+) in both Pink1 WT and Pink1 KO mice [27]. These findings suggest that inhibiting PHLPP may confer neuroprotection in a PD model. However, a major limitation of that study is its use of primary cortical neurons instead of the SN neurons to investigate the role of PHLPP in PD, highlighting the need for new evidence that specifically addresses PHLPP’s effects in the SN, which is crucial for understanding the pathology of PD.

As a vital isoform of PHLPP, PHLPP1 has been demonstrated to exert deleterious effects on neurons both in vivo and in vitro. In our study, we demonstrated that PHLPP1 deficiency significantly reduced dopaminergic neuronal death in SN of the KO-MPTP group compared to the WT group and consequently ameliorated MPTP-induced neurobehavioral dysfunction. This provides direct evidence linking PHLPP1 to the pathophysiology of PD.

Furthering this exploration, Jackson et al. studied the impact of the two PHLPP1 isoforms on neuronal function and found that PHLPP1α had a more pronounced influence on the Akt signaling pathway which is pivotal for neuroprotection. Their results indicated that knockdown of PHLPP1α/β led to increased Akt phosphorylation and expression without affecting PKC phosphorylation or expression, indicating neuroprotective effects. In contrast, knockdown of PHLPP1β/SCOP alone resulted in decreased Akt phosphorylation, as well as reduced PKCα phosphorylation and expression [28]. These findings point to the differential roles of PHLPP1 isoforms in SN neurons in the context of PD, indicating a need for further investigation to fully elucidate these distinct effects and their implications for PD pathology. Though the present study did not test changes of AKT and related markers on this signaling pathway, our results from knockout of PHLPP indicate that apoptosis, neuroinflammation related markers are all downregulated. These might reflect the neuroprotective effect of PHLPP knockout, potentially through the AKT signaling pathway.

In both the histology and Western blotting experiments, TH was found to decrease in the MPTP model and this decrease was reversed in the PHLPP knockout mouse model. TH is the speed limiting enzyme for producing dopamin**e** [20, 29]. The preservation of TH after PHLPP knockout suggests increased content of dopamine in the substantia nigra. This assumption was confirmed by behavioural tests. In the open field test, walking distance, mean walking speed, rearing time and number of squares the mice crossed were all increased in the PHLPP-knockout group compared to the MPTP model group. In the meantime, the time to reach the bottom of the pole was increased compared to the model group, further consolidating the assumption that dopamine content in the substantia nigra might be preserved in PHLPP knockout mice.

In 1988, a study by McGeer et al. showed the presence of neuroinflammation in PD brains by identifying activated microglia in the SN of postmortem patients [30]. Notably, SN has the highest microglial cell density among brain regions, indicating a significant role of this type of immune cells in the physiological condition. Neuronal loss in PD is primarily driven by neuroinflammatory mechanisms characterized by the activation of the innate immune system which includes microglia and their secreted mediators surrounding degenerating neurons.

Microglia play a pivotal role in inflammatory response by secreting pro-inflammatory cytokines, such as IL-1β, IL-6, IL-12, IL-17, IL-18, IL-23, TNFα, IFNγ, as well as nitric oxide (NO). They also exhibit phenotypic markers indicative of activation, including major histocompatibility complex class II (MHC II), inducible nitric oxide synthase (iNOS), CD86, cyclooxygenase-2 (COX2), reactive oxygen species (ROS), and prostaglandin E2 (PGE2) [7, 31]. Oxidative stress harbours reactive microgliosis which is an important etiopathology in PD fostering neurodegeneration. This oxidative stress is due to the ROS and/or RNS produced by the microglia itself. Sometimes these species can be produced by the mitochondrial respiratory chain inhibition by MPP+, an active metabolite of MPTP. Sinha et al. has shown increase in ROS at day7 after MPTP administration [21]. This oxidative stress promotes the proinflammatory activity of microglia releasing proinflammatory cytokines like Il-1β, TNF-α, Il-6 and supresses the anti-inflammatory action. In addition, multiple studies have shown that Il-1β and TNF-α levels are significantly elevated in the PD model induced by intraperitoneal injection of MPTP [3, 32–35], which is consistent with our results.

To further study the role of PHLPP1 in neuroinflammation observed in PD, the impact of PHLPP1 on levels of inflammatory cytokines in SN of a MPTP model was assessed. Our findings showed that in KO-MPTP mice, levels of pro-inflammatory mediators, such as IL-1β, IL-6, TNF-α, and iNOS were significantly downregulated, while levels of the anti-inflammatory cytokines like TGF-β was upregulated compared to WT-MPTP mice. These findings suggest that PHLPP1 influences the inflammatory profile of microglia, thereby altering the inflammatory environment in the SN. Increased TGF-β may synergize with the decrease level of inflammatory cytokines to attenuate neural injury. This modulation of neuroinflammation by PHLPP1 is likely a contributing factor to the protection against dopaminergic neuronal death, highlighting its potential to become a therapeutic target in the management of PD and its associated neuroinflammatory processes.

In PD, neuroinflammation is characterized by the activation of microglia, which results in increased secretion of interleukin-1β (IL-1β). A primary driver of IL-1β secretion from these microglia is the NLRP3 inflammasome. Notably, it is activated by aggregated α-synuclein (SNCA), which is closely associated with the pathogenesis of various synucleinopathies, particularly PD [36]. This inflammasome is composed of three key components [37–40]: (1) the intracellular pattern recognition receptor known as NLRP3 (NACHT, LRR, and PYD domain-containing protein 3); (2) the adaptor protein apoptosis-associated speck-like protein containing a caspase activation and recruitment domain (ASC); and (3) the cysteine-aspartate protease-1 (caspase-1). Upon stimulation of microglial Toll-like receptors by α-synuclein (signaling I), IL-1β is initially translated into an inactive precursor (pro-IL-1β, 31 kDa), and subsequently cleaved into its active 17 kDa form by the NLRP3 inflammasome-activated caspase-1 (signaling II). Elevated levels of the pro-inflammatory cytokine IL-1β, produced by microglia in the central nervous system (CNS), were observed in both PD patients and experimental animal models, correlating with the extent of inflammation and neuronal injury [41, 42]. Studies using gene-deletion mouse models have demonstrated that NLRP3-PYCARD-CASP1-mediated maturation of IL-1β is critical for MPTP-induced neurodegeneration as nlrp−/− mice exhibited less severe neurodegeneration compared to WT mice [43].

In our study, we found that PHLPP1 deficiency led to reduced activation of IL-1β and caspase-1 in the MPTP model compared to WT-MPTP mice. Moreover, PHLPP1 was shown to bind to NLRP3 in SN of WT-MPTP mice. These observations suggest that PHLPP1 may interact with caspase-1, a component of the NLRP3 inflammasome, thereby influencing IL-1β secretion by modulating caspase-1 levels in SN of MPTP model mice. The NF-κB signaling pathway plays an essential role in neuroinflammation [3, 44]. Its expression or activation has been impacted by many factors including proinflammatory cytokines like IL-1β, oxidative stress, DNA damage, bacterial and even viral products. The present study found that PHLPP knockout significantly mitigated apoptosis, oxidative stress induced by MPTP, and even the proinflammatory markers like IL-1β and NLRP3. These suggest that PHLPP might indirectly activate the NF-κB signaling pathway and alleviate neuroinflammation.

In addition, findings by Pike et al. indicate a discrepancy in this mechanism, revealing that caspase-1 inhibition does not appear to block IL-1β secretion by primary human microglia [45], in contrast to primary mouse microglia. This raises concerns about the specific role of PHLPP1 in NLRP3-mediated IL-1β secretion by human microglia within SN. Investigating this effect in human microglia is crucial to understand the broader implications of PHLPP1 in neuroinflammation and its potential to become a therapeutic target in PD.

Our study has its own limitations. First of all, this study only selected male mice and received 4 injections of MPTP within 1 day, which may result in different findings from previous studies. Secondly, PHLPP1 was knocked out in this mouse model, but which subtype of PHLPP1 is mainly responsible for the behavioural phenotype is not examined. These need to be addressed in future studies.

In summary, the present study provides compelling evidence on the role of PHLPP1 in inflammation-associated neurodegeneration within SN in a PD mouse model. Our results demonstrate that PHLPP1 deficiency protects neurons in the SN from MPTP-induced dopaminergic cell death and leads to improved neurological outcomes. This protective effect appears to involve the modulation of NLRP3-PYCARD-CASP1-mediated IL-1β secretion, and the regulation of other pro-inflammatory cytokines expressed by microglia.

## Conclusions

Overall, our data offer phenotypic evidence of the pathological mechanisms involving PHLPP1 in PD, underpinning its potential as a drug target for mitigating neuroinflammation and dopaminergic neuronal loss in this debilitating disease. Further understanding of the role of PHLPP1 could facilitate the development of new interventions that aim to slow or prevent the progression of neurodegeneration in PD.

## Data Availability

No datasets were generated or analysed during the current study.

## References

[1] Kalia LV, Lang AE. Parkinson’s Disease Lancet. 2015;386:896–912. 25904081 10.1016/S0140-6736(14)61393-3

[2] Mitra S, Chakrabarti N, Dutta SS, Ray S, Bhattacharya P, Sinha P, Bhattacharyya A. Gender-specific brain regional variation of neurons, endogenous estrogen, neuroinflammation and glial cells during rotenone-induced mouse model of parkinson’s disease. Neuroscience. 2015;292:46–70. 25592425 10.1016/j.neuroscience.2014.12.052

[3] Mitra S, Ghosh N, Sinha P, Chakrabarti N, Bhattacharyya A. Alteration in nuclear Factor-KappaB pathway and functionality of Estrogen via receptors promote neuroinflammation in frontal cortex after 1-Methyl-4-Phenyl-1,2,3,6-Tetrahydropyridine treatment. Sci Rep. 2015;5:13949. 26365888 10.1038/srep13949 PMC4568517

[4] Wang W, Song N, Jia F, Tang T, Bao W, Zuo C, Xie J, Jiang H. Genomic DNA levels of mutant Alpha-synuclein correlate with Non-Motor symptoms in an A53T parkinson’s disease mouse model. Neurochem Int. 2018;114:71–9. 29355568 10.1016/j.neuint.2018.01.006

[5] Fitzgerald E, Murphy S, Martinson HA. Alpha-synuclein pathology and the role of the microbiota in parkinson’s disease. Front Neurosci. 2019;13:369. 31068777 10.3389/fnins.2019.00369 PMC6491838

[6] Xu L, Pu J. Alpha-Synuclein in Parkinson’s Disease: From Pathogenetic Dysfunction to Potential Clinical Application. Park. Dis. 2016;2016:1720621. 10.1155/2016/1720621 PMC5005546 27610264

[7] Shao W, et al. Suppression of neuroinflammation by astrocytic dopamine D2 receptors via alphaB-crystallin. Nature. 2013;494:90–4. 23242137 10.1038/nature11748

[8] Yadav SK, Rai SN, Singh SP. Mucuna pruriens reduces inducible nitric oxide synthase expression in parkinsonian mice model. J Chem Neuroanat. 2017;80:1–10. 27919828 10.1016/j.jchemneu.2016.11.009

[9] Rai SN, Yadav SK, Singh D, Singh SP. Ursolic acid attenuates oxidative stress in nigrostriatal tissue and improves neurobehavioral activity in MPTP-induced parkinsonian mouse model. J Chem Neuroanat. 2016;71:41–9. 26686287 10.1016/j.jchemneu.2015.12.002

[10] Prakash J, Chouhan S, Yadav SK, Westfall S, Rai SN, Singh SP. Withania somnifera alleviates parkinsonian phenotypes by inhibiting apoptotic pathways in dopaminergic neurons. Neurochem Res. 2014;39(12):2527–36. 25403619 10.1007/s11064-014-1443-7

[11] Ramakrishna K, Nalla LV, Naresh D, Venkateswarlu K, Viswanadh MK, Nalluri BN, Chakravarthy G, Duguluri S, Singh P, Rai SN, Kumar A, Singh V, Singh SK. WNT-β Catenin signaling as a potential therapeutic target for neurodegenerative diseases: current status and future perspective. Diseases. 2023;11(3):89. 37489441 10.3390/diseases11030089 PMC10366863

[12] Miyamoto S, Purcell NH, Smith JM, Gao T, Whittaker R, Huang K, Castillo R, Glembotski CC, Sussman MA, Newton AC, Heller Brown J. PHLPP-1 negatively regulates Akt activity and survival in the heart. Circ Res. 2010;107(4):476–84. 20576936 10.1161/CIRCRESAHA.109.215020 PMC2957297

[13] Warfel NA, Niederst M, Stevens MW, Brennan PM, Frame MC, Newton AC. Mislocalization of the E3 ligase, β-transducin repeat-containing protein 1 (β-TrCP1), in glioblastoma uncouples negative feedback between the pleckstrin homology domain leucine-rich repeat protein phosphatase 1 (PHLPP1) and Akt. J Biol Chem. 2011;286(22):19777–88. 21454620 10.1074/jbc.M111.237081 PMC3103356

[14] Chen B, van Winkle JA, Lyden PD, Brown JH, Purcell NH. PHLPP1 gene deletion protects the brain from ischemic injury. J Cereb Blood Flow Metab. 2013;33(2):196–204. 23072745 10.1038/jcbfm.2012.150 PMC3564187

[15] Jackson TC, Verrier JD, Drabek T, Janesko-Feldman K, Gillespie DG, Uray T, Dezfulian C, Clark RS, Bayir H, Jackson EK, Kochanek PM. Pharmacological Inhibition of pleckstrin homology domain leucine-rich repeat protein phosphatase is neuroprotective: differential effects on astrocytes s. J Pharmacol Exp Ther. 2013;347(2):516–28. 24023368 10.1124/jpet.113.206888 PMC3807060

[16] Shimizu K, Phan T, Mansuy IM, Storm DR. Proteolytic degradation of SCOP in the hippocampus contributes to activation of MAP kinase and memory. Cell. 2007;128(6):1219–29. 17382888 10.1016/j.cell.2006.12.047 PMC1899088

[17] Saito K-I, Elcet JS, Hamos JE, Nixon RA. Widespread activation of calciumactivated neutral proteinase (calpain) in the brain in alzheimer disease: a potential molecular basis for neuronal degeneration. Proc Natl Acad Sci U S A. 1993;90(7):2628–32. 8464868 10.1073/pnas.90.7.2628 PMC46148

[18] Saavedra A, García-Martínez JM, Xifro X, Giralt A, Torres-Peraza JF, Canals JM, Díaz-Hernandez M, Lucas JJ, Alberch J, P´erez-Navarro E. PH domain leucine-rich repeat protein phosphatase 1 contributes to maintain the activation of the PI3K/Akt pro-survival pathway in huntington’s disease striatum. Cell Death Differ. 2010;17(2):324–35. 19745829 10.1038/cdd.2009.127

[19] Jackson-Lewis V, Przedborski S. Protocol for the MPTP mouse model of parkinson’s disease. Nat Protoc. 2007;2(1):141–51. 17401348 10.1038/nprot.2006.342

[20] Rai SN, Singh P. Advancement in the modelling and therapeutics of parkinson’s disease. J Chem Neuroanat. 2020;104:101752. 31996329 10.1016/j.jchemneu.2020.101752

[21] Sinha P, Chakrabarti N, Ghosh N, Mitra S, Dalui S, Bhattacharyya A. Alterations of thyroidal status in brain regions and hypothalamo-pituitary-blood-thyroid-axis associated with dopaminergic depletion in substantia Nigra and ROS formation in different brain regions after MPTP treatment in adult male mice. Brain Res Bull. 2020;156:131–40. 31891753 10.1016/j.brainresbull.2019.12.013

[22] Huang Y, Liu Z, Wang XQ, Qiu YH, Peng YP. A dysfunction of CD4 + T lymphocytes in peripheral immune system of parkinson’s disease model mice. Zhongguo Ying Yong Sheng Li Xue Za Zhi. 2014;30(6):567–76. 26016368

[23] Wu DD, Huang L, Zhang L, Wu LY, Li YC, Feng L. LLDT-67 attenuates MPTP-induced neurotoxicity in mice by up-regulating NGF expression. Acta Pharmacol Sin. 2012;33(9):1187–94. 22941283 10.1038/aps.2012.88 PMC4003115

[24] Luster AD, Alon R, von Andrian UH. Immune cell migration in inflammation: present and future therapeutic targets. Nat Immunol. 2005;6(12):1182–90. 16369557 10.1038/ni1275

[25] Hunot S, Vila M, Teismann P, Davis RJ, Hirsch EC, Przedborski S, Rakic P, Flavell RA. JNK-mediated induction of cyclooxygenase 2 is required for neurodegeneration in a mouse model of parkinson’s disease. Proc Natl Acad Sci U S A. 2004;101(2):665–70. 14704277 10.1073/pnas.0307453101 PMC327205

[26] Livak KJ, Schmittgen TD. Analysis of relative gene expression data using real-time quantitative PCR and the 2(-Delta Delta C(T)) Method. Methods (San Diego, Calif.), 2001;25(4):402–408. 10.1006/meth.2001.1262 11846609

[27] Boonying W, Joselin A, Huang E, Qu D, Safarpour F, Iyirhiaro GO, Gonzalez YR, Callaghan SM, Slack RS, Figeys D, Chung YH, Park DS. Pink1 regulates FKBP5 interaction with AKT/PHLPP and protects neurons from neurotoxin stress induced by MPP+. J Neurochem. 2019;150(3):312–29. 30734931 10.1111/jnc.14683

[28] Jackson TC, Verrier JD, Semple-Rowland S, Kumar A, Foster TC. PHLPP1 splice variants differentially regulate AKT and PKCα signaling in hippocampal neurons: characterization of PHLPP proteins in the adult hippocampus. J Neurochem. 2010;115(4):941–55. 20819118 10.1111/j.1471-4159.2010.06984.x PMC3730267

[29] El-Latif AMA, Rabie MA, Sayed RH, Fattah MAAE, Kenawy SA. Inosine attenuates rotenone-induced parkinson’s disease in rats by alleviating the imbalance between autophagy and apoptosis. Drug Dev Res. 2023;84(6):1159–74. 37170799 10.1002/ddr.22077

[30] McGeer PL, Itagaki S, Boyes BE, McGeer EG. Reactive microglia are positive for HLA-DR in the substantia Nigra of parkinson’s and alzheimer’s disease brains. Neurology. 1988;38:1285–91. 3399080 10.1212/wnl.38.8.1285

[31] Rogers J, Mastroeni D, Leonard B, Joyce J, Grover A. Neuroinflammation in alzheimer’s disease and parkinson’s disease: are microglia pathogenic in either disorder? Int Rev Neurobiol. 2007;82:235–46. 17678964 10.1016/S0074-7742(07)82012-5

[32] Huang D, Wang Z, Tong J, Wang M, Wang J, Xu J, Bai X, Li H, Huang Y, Wu Y, Ma Y, Yu M, Huang F. Long-term changes in the nigrostriatal pathway in the MPTP mouse model of parkinson’s disease. Neuroscience. 2018;369:303–13. 29196026 10.1016/j.neuroscience.2017.11.041

[33] Liddelow, S. A., Guttenplan, K. A., Clarke, L. E., Bennett, F. C., Bohlen, C. J.,Schirmer, L., Bennett, M. L., Münch, A. E., Chung, W. S., Peterson, T. C., Wilton,D. K., Frouin, A., Napier, B. A., Panicker, N., Kumar, M., Buckwalter, M. S., Rowitch,D. H., Dawson, V. L., Dawson, T. M., Stevens, B., … Barres, B. A. Neurotoxic reactive astrocytes are induced by activated microglia. Nature, 2017;541(7638):481–487. 10.1038/nature21029 PMC5404890 28099414

[34] Mitra S, Chakrabarti N, Bhattacharyya A. Differential regional expression patterns of α-synuclein, TNF-α, and IL-1β; and variable status of dopaminergic neurotoxicity in mouse brain after Paraquat treatment. J Neuroinflamm. 2011;8:163. 10.1186/1742-2094-8-163 PMC3247140 22112368

[35] Qin X, Wang S, Huang J, Hu B, Yang X, Liang L, Zhou R, Huang W. Rhein alleviates MPTP-induced parkinson’s disease by suppressing neuroinflammation via MAPK/IκB pathway. Front NeuroSci. 2024;18:1396345. 38933815 10.3389/fnins.2024.1396345 PMC11202316

[36] Codolo G, Plotegher N, Pozzobon T, et al. Triggering of inflammasome by aggregated alpha- synuclein, an inflammatory response in synucleinopathies. PLoS ONE. 2013;8:e55375. 23383169 10.1371/journal.pone.0055375 PMC3561263

[37] Gaidt MM, Hornung V. The NLRP3 inflammasome renders cell death Pro-inflammatory. J Mol Biol. 2018;430(2):133–41. 29203171 10.1016/j.jmb.2017.11.013

[38] Schroder K, Tschopp J. The inflammasomes. Cell. 2010;140(6):821–32. 20303873 10.1016/j.cell.2010.01.040

[39] Swanson KV, Deng M, Ting JP. The NLRP3 inflammasome: molecular activation and regulation to therapeutics. Nat Rev Immunol. 2019;19(8):477–89. 31036962 10.1038/s41577-019-0165-0 PMC7807242

[40] Gritsenko A, Green JP, Brough D, Lopez-Castejon G. Mechanisms of NLRP3 priming in inflammaging and age related diseases. Cytokine Growth Factor Rev. 2020;55:15–25. 32883606 10.1016/j.cytogfr.2020.08.003 PMC7571497

[41] Athauda D, Foltynie T. The ongoing pursuit of neuroprotective therapies in Parkinson disease. Nat Rev Neurol. 2015;11:25–40. 25447485 10.1038/nrneurol.2014.226

[42] Koprich JB, Kalia LV, Brotchie JM. Animal models of sncaopathy for Parkinson disease drug development. Nat Rev Neurosci. 2017;18:515–29. 28747776 10.1038/nrn.2017.75

[43] Ding B, Lin C, Liu Q, He Y, Ruganzu JB, Jin H, Peng X, Ji S, Ma Y, Yang W. Tanshinone IIA attenuates neuroinflammation via inhibiting RAGE/NF-κB signaling pathway in vivo and in vitro. J Neuroinflamm. 2020;17(1):302. 10.1186/s12974-020-01981-4 PMC7559789 33054814

[44] An Y, Jiang W, Liu L, Wang X, Ding C, Tian Z, Zhou R. Dopamine controls systemic inflammation through Inhibition of NLRP3 inflammasome. Cell. 2015;160(1–2):62–73. 25594175 10.1016/j.cell.2014.11.047

[45] Pike AF, Varanita T, Herrebout MAC, Plug BC, Kole J, Musters RJP, Teunissen CE, Hoozemans JJM, Bubacco L, Veerhuis R. α-Synuclein evokes NLRP3 inflammasome-mediated IL-1β secretion from primary human microglia. Glia. 2021;69(6):1413–28. 33506583 10.1002/glia.23970 PMC8247862

